# A Bayesian Model for Estimation of Virus Reduction in Potable Reuse Treatment Trains and Quantitative Microbial Risk Assessment of Waterborne Viral Pathogens

**DOI:** 10.1111/risa.70343

**Published:** 2026-08-29

**Authors:** Jack F. Schijven, Peter F. M. Teunis, Walter Q. Betancourt

**Affiliations:** ^1^ Environmental Hydrogeology Group, Geosciences Utrecht University Utrecht The Netherlands; ^2^ Center for Global Safe WASH Rollins School of Public Health Emory University Atlanta Georgia USA; ^3^ Department of Environmental Science College of Agriculture Life and Environmental Sciences University of Arizona Tucson Arizona USA

**Keywords:** advanced treatment, log reduction model, potable reuse, QMRA, viruses

## Abstract

Understanding virus occurrence and reduction at advanced treatment facilities for potable water reuse constitutes a high‐priority research need to protect human health and to enhance available water supply alternatives. The objectives were to (1) determine log reduction values (LRVs) of 15 viruses by advanced wastewater treatment; (2) evaluate suitability of the final wastewater effluent for potable reuse; (3) evaluate viruses or groups of viruses as indicators of wastewater treatment efficiency. Reduction data of seven human enteric and eight surrogate viruses were obtained from three potable reuse facilities. LRVs were estimated using a Bayesian model that can handle nondetects and identifies groups of viruses with similar LRVs. Quantitative microbial risk assessments were conducted for adenoviruses, enteroviruses, and noroviruses GI and GII. Mean total LRVs ranged from 5.4 to 11 log_10._ Required mean total LRVs for the four pathogenic viruses ranged from 11.3 to 13.4 log_10_. Both male‐specific and somatic coliphages, detected by classical enumeration of infectious virions, are recommended as indicator viruses. Of the pathogenic viruses, adenovirus was found to be the most effective indicator for virus reduction. At all facilities, infection risks were higher than 10^−4^ per person per year, implying that the finished water may not comply with existing safety standards. Infection risks may have been overestimated by 2–4 log_10_ because only a fraction of the detected virus was infectious. Nevertheless, achieved LRVs were still too low and given the uncertainty on infectious virus fraction, one may accept overestimation of risks to stay on the safe side.

## 1 Introduction

Advanced water treatment processes are indispensable to mitigate microbial and chemical contaminants present in recycled waters for potable reuse. Enteric viruses involved in waterborne disease represent a significant risk in water reuse applications due to their high concentrations in untreated wastewater, their small size, and properties that render them resistant to reduction by water treatment technologies (Armanious and Mezzenga 2022; Gerba et al. 2017, Gerba et al. 2018; Lanrewaju et al. 2022; Nappier et al. 2018; Soller et al. 2017; Zhong et al. 2017). Understanding virus occurrence and reduction at full‐scale advanced treatment processes for potable reuse applications constitutes a high‐priority research need to protect human health and to enhance the evaluation of available water supply alternatives in the United States (NRC 2012). In raw wastewater, the incidence of viruses is influenced by the size of the human population in the catchment, while virus reduction is influenced by treatment efficiency (Gerba et al. 2017; Schmitz et al. 2016). Therefore, the most effective virus‐reduction technologies must be implemented during wastewater treatment to minimize the risks associated with viruses in effluent waters for discharge or for reuse purposes (Chaudhry et al. 2017; Gerba et al. 2017; Harwood et al. 2005; Rose et al. 2004; Scott et al. 2003; Soller et al. 2003_)__._

The U.S Environmental Protection Agency regulatory guidelines for water reuse practices describe treatment technologies and monitoring requirements to assist states and nations in developing water reuse programs and regulations that are protective of public and environmental health (U.S. Environmental Protection Agency 2012). State and local agencies are responsible for setting potable reuse standards mostly in terms of log reduction values (LRVs) to not exceed a risk of infection of 10^−4^ per person per year (Chaudhry et al. 2017). Required LRVs vary widely from 8 log_10_ (Texas Commission on Environmental Quality 2022) to 12 log_10_ (California Department of Public Health 2014) and even up to 20 log_10_ (California State Water Resources Control Board Division of Drinking Water 2021). States such as Arizona, Colorado, and Florida have developed their own guidelines on the log_10_ reduction credits proposed in the California and Texas regulatory frameworks. Apparently, there is a wide range in required LRVs, and harmonization is desired (Jahne et al. 2024).

In a study (Betancourt 2023) at three facilities that apply advanced water purification technologies for removal of contaminants from recycled water, samples were collected at each stage of treatment and analyzed for seven different enteric viruses (adenovirus, aichivirus, bocavirus, enterovirus, norovirus GI, norovirus GII, and reovirus) to determine their log_10_ reduction values. In addition, the samples were also analyzed for surrogate viruses, including culturable somatic and male‐specific coliphages, CRESS viruses (Circular Rep‐encoding ssDNA viruses, including Hudisavirus, WCDV‐ 1, 2, and 3 (Rosario et al. 2019)), Pepper Mild Mottle Virus (PMMoV), and human gut associated bacteriophage (crAssphage) to investigate their potential as process indicators of enteric virus reduction. These viruses, frequently found upstream of advanced treatment trains, have also well‐documented environmental persistence and resilience to wastewater treatment processes (Betancourt 2023). All viruses were detected by means of digital PCR, except for the somatic and male‐specific coliphages, which were detected by classical plaque counting. Inevitably, after every successive treatment step, relatively more nondetects (zero counts) were collected. Availability of virus concentrations at each step of a treatment chain for 15 viruses at three facilities for advanced wastewater treatment makes the data set unique for assessing variation in LRV by virus and treatment.

The aims of the study presented here were:

1. To determine LRVs of each of the studied viruses for each studied wastewater treatment.

To that aim, a Bayesian LRV model was developed that can handle nondetects (censored data). The effect of many nondetects in the later stages of treatment on uncertainty of LRVs must be dealt with appropriately. Because the selected viruses were all indigenous to municipal wastewater, the LRV model grouped viruses with similar LRVs to attempt generalization of LRVs across virus groups and characterize variation within these groups. Estimating log reduction by a treatment for a single virus will be very uncertain and, in some cases, impossible if a large proportion of the concentration data are nondetects. When all concentration data are nondetects, only a minimum log reduction may be estimated. PCR enables the detection of a plethora of different viruses, in our case 15, from the same sample. It is plausible that a number of those viruses are similarly reduced in concentration, and, therefore, a group reduction may be estimated incorporating the variation in log reduction among the viruses in the group.

2. To evaluate whether the final wastewater effluent was suitable for potable reuse.

The LRV model was applied in a quantitative microbial risk assessment (QMRA) framework to evaluate whether the finished water met requirements for direct potable reuse regarding exposure to adenovirus, enterovirus, and noroviruses GI and GII.

3. To evaluate viruses or groups of viruses as indicators of wastewater treatment efficiency.

This was done by comparing LRVs and risks. Risks for the four selected pathogenic viruses were estimated using LRVs of those viruses, as well as those of the virus groups to which the pathogenic virus was classified and those of surrogate viruses. Surrogate viruses are the best indicator for reduction of the pathogenic viruses that belong to the same group.

## 2 Methods

### 2.1 Facilities and Sampling Points

Three facilities (A, B, and D) that apply advanced wastewater treatment were investigated. Table A1 lists the treatment chains of these facilities. Details are available in Betancourt (2023).

Facility A takes tertiary effluent from a conventional water reclamation treatment plant and further treats it through ozonation, membrane ultrafiltration, reverse osmosis, and ultraviolet photolysis. Sampling points included raw wastewater (RAW), tertiary effluent (TER), reverse osmosis permeate (ROP), and UV permeate (UVP). A series of 14 samples were collected monthly from June 2021 until July 2022.

Facility B is an operational underground storage and recovery facility. Sampling points were raw wastewater (RAW), tertiary effluent (TER), and recharged groundwater (RGW) from two extraction wells. A series of nine samples were collected from April 2021 until June 2022.

Facility D includes a treatment train based on coagulation/filtration/sedimentation, ozonation, biofiltration (BAF), granular activated carbon (GAC) adsorption, and UV disinfection, followed by managed aquifer recharge (Hogard et al. 2021). Sampling points included raw wastewater (RAW), secondary effluent (SEC), settled water (SETTLW), ozone effluent (OZONE), biological activated carbon effluent (BAC), granular activated carbon effluent (GAC), UVP, and recharge groundwater from soil aquifer treatment (MW_SAT). A series of only three samples were collected (February, April, and August 2022).

### 2.2 Virus Detection

Two methods were used for virus concentration from the different sampling sites. The recovery and concentration of viruses from raw wastewater was accomplished by stacked membrane filtration followed by centrifugal ultrafiltration using a method previously described (Betancourt et al. 2021). The VIRADEL (VIRus ADsorption‐ELution) method based on the adsorption of viruses to electro‐positively charged NanoCeram filters (Argonide Corporation, Sanford, FL) was used for primary concentration of viruses from large volumes (>10 L) followed by virus elution from the filters using 350 mL of sodium polyphosphate buffer (pH 9.5). Aliquots of 50–100 mL were allocated for analysis of culturable male‐specific (F+) and somatic coliphages using a modification of the single agar layer method to process large water sample volumes as described elsewhere (Betancourt 2023; McMinn et al. 2018). The rest of the virus eluate was used for secondary concentration by centrifugal ultrafiltration via Centricon Plus‐70 centrifugal ultrafilters (100 kDa cutoff; Millipore, Billerica, MA) following the manufacturer's instructions. Water concentrates were used immediately for nuclease treatment and viral nucleic acid extraction as previously described (Betancourt 2023). Absolute quantification of nuclease‐protected virus genomes was accomplished by digital PCR on the QIAcuity Digital PCR system (QIAGEN, Valencia, CA) using 26k 24‐well Nanoplates. The concentrations of primers and probes, as well as amplification conditions for virus detection and quantification, are described in detail elsewhere (Betancourt 2023).

### 2.3 Raw Data Advanced Treatment

Table A1 provides the list of studied viruses, numbers of samples, numbers of nondetects, and numbers of samples that did not produce results for a particular virus. The raw data, that is, genome copies or plaque‐forming units and sample volumes, are available in spreadsheet Complete_Virusdata.xlsx (Supplementary MateriaI).

### 2.4 Raw Data for Sensitivity of Detection

Raw data for sensitivity of detection in terms of digital PCR (dPCR) genome copies and sample volumes were collected from four samples at facility B for coxsackievrius B5 (CV‐B5), human adenovirus 4 (HAdV‐4), murine norovirus (MNV), porcine parvovirus (PPV), and reovirus T3D in tertiary effluent (TER), recharge groundwater, and permeate streams of ultrafiltration and nanofiltration membrane elements that form part of an engineering integrated membrane system for on‐site potable reuse (Souza‐Chaves et al. 2022). The sensitivity of a detection method is the probability of detecting an organism when it is known to be present (Teunis and Schijven 2019). Note that sensitivity of detection is the same as the often‐used recovery efficiency. Water samples were sourced from facility B, which generates disinfected tertiary recycled water for soil‐aquifer treatment and for in‐house advanced treatment via ultrafiltration‐nanofiltration for on‐site nonpotable reuse. The samples were spiked with 6×10^5^–2×10^6^ genome copies of coxsackievirus B5, human adenovirus 4, murine norovirus, porcine parvovirus (PPV), and reovirus T3D. The raw data are available in the spreadsheet CompleteRecoveriesWRF.xlsx (Supplementary Material). Raw wastewater was not included because of the high load of viruses already present in these matrices.

### 2.5 LRV Model

In addition to the estimation of reductions per virus and water treatment, two groups of viruses were identified and characterized by their reductions per treatment.

The LRV model was coded in JAGS (version 4.3.0, Plummer 2017). Genome copy counts from dPCR in a sample were assumed to be Poisson distributed:

(1) $$nobs_{s,v,o}\;P\left[e^{x\mu_{s,v,o}}*V_{s,v,o}\right],$$

where *nobs* is the genome copy count, *P* is the Poisson distribution, *x*μ is the *log* mean concentration, and *V* is the sample volume (L) per stage (*s*), virus (*v*), and observation (*o*).

The first stage represents untreated sewage, and the subsequent stages represent the effluents of the consecutive treatment steps.

For each stage *s > 1*, each virus *v*, and each observation *o*, the pairwise virus log reductions *robs* were determined:

(2) $$robs_{s-1,v,o}=x\mu_{s-1,v,o}\;-x\mu_{s,v,o},\;$$

where the log reduction *robs_s‐1,v,o_* again was assumed to have a normal distribution:

(3) $$robs_{s-1,v,0}\;N\left[red_{s-1,v},o\tau_{s-1,v}\right],$$

with *N* for normal distribution, mean log reduction *red_s‐1,v_* and precision o*τ_s‐1,v_* per virus and stage.

To study grouping of log reductions, the log reduction per treatment and per virus, *rred_s_*_‐1,v_ was modeled as a mixture of normal distributions (function *dnormmix* in JAGS). Because the numbers of viruses and treatments are limited, the number of components in the mixture was limited to two (*ngroup* = 2).

(4) $$rred_{s-1,v}\;dnormmix\left[\mu_{s-1,1:nrgroup},r\tau_{s-1,1:ngroup},r\pi_{s-1,v,1:ngroup}\right],\;$$

where *r_µs‐1,1;ngroup_* is the mean reduction per treatment and group, *rτ_s‐1,1;ngroup_* is the precision per treatment and group, and *rπ_s‐1,1:ngroup_* is the probability that a virus belongs in treatment group 1 (and with probability 1‐*rπ_s‐1,1:ngroup_* in treatment group 2). These probabilities were assumed to be Dirichlet distributed (or, in this case, with *ngroup* = 2: beta distributed).

(5) $$r\pi_{s-1,v,1:ngroup}\;ddirch\left[\alpha_{s-1,1:ngroup}\right],$$

where the grouping parameter α_s‐1,1:ngroup_ was set to the small value of 10^−1^, where the Dirichlet distribution (*ddirch*) is U‐shaped to favor distinct grouping.

Note that normally distributed *rred* allows negative values (i.e., increase instead of decrease in virus concentration). In order to prevent negative virus reduction values, *rred_s‐1,v_* a transformation was applied to obtain a log reduction:

$$\mathrm{re}d_{s-1,v}=\mathrm{rre}d_{s-1,v}\;\text{if}\;\mathrm{rre}d_{s-1,v}>\mathrm{inflec}\;\text{and}$$

(6) $$red_{s-1,v}=inflec*exp\left(rred_{s-1,v}\;-inflec\right)\;\text{if}\;rred_{s-1,v}\leq inflec,$$

with parameter *inflec* = 0.2 which causes *red_s‐1,v_* to smoothly approach zero.

Prior distributions for means and precisions of *x* (log concentration), *r* (group reduction), and *o* (virus reduction):

(7) $$x\mu_{1,v,o}\;N\left[x\mu hyp_{v,1},x\mu hyp_{v,2}\right]$$

(8) $$r\mu_{s,g}\;N\left[r\mu hyp_{s,g,1},r\mu hyp_{s,g,2}\right]$$

(9) $$x\tau_{s,v,}\;Gamma\left[x\tau hyp_{s,1},x\tau hyp_{s,2}\right]$$

(10) $$r\tau_{s,g}\;Gamma\left[r\tau hyp_{s,g,1},r\tau hyp_{s,g,2}\right]$$

(11) $$o\tau_{s,v}\;Gamma\left[o\tau hyp_{s,1},o\tau hyp_{s,2}\right]$$

with Gamma denoting the Gamma distribution.

**FIGURE A1:**
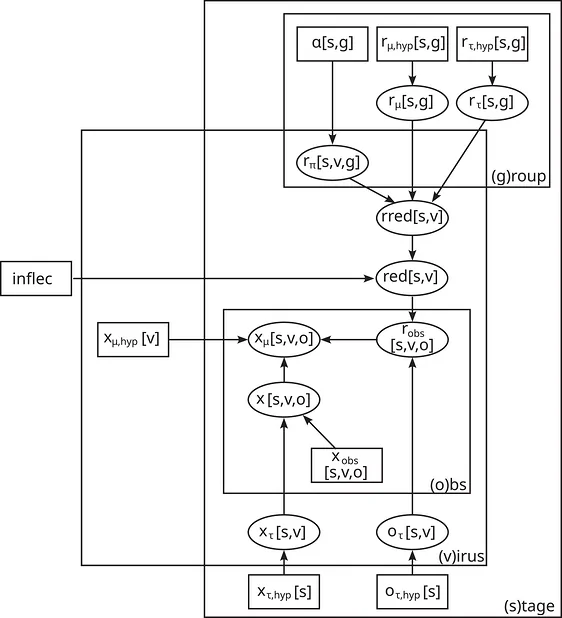
Directed acyclic graph of the JAGS LRV model for estimating virus reductions in groups of similar reduction and reduction per virus per treatment. See Equations (1–11).

Figure A1 gives an overview of the LRV model in a directed acyclic graph.

To facilitate identification of virus groups, the variance in log reduction of each group was assumed to be narrow in order to increase the contrast between groups and to avoid groups with a very wide range in posterior log reductions that would be meaningless (trivial solution). At the same time, a wide prior for the means of the group distributions was set in order to avoid restriction of the location of log reduction groups.

Hyperprior vector *xμhyp*, vector for the log mean concentration and its precision at stage = 1 for each virus, was set to c(4, 10^−3^).

Hyperprior vector *rμhyp*, vector for the group log mean reductions and their precisions, was set to c(1.0,1.0). Hyperprior vectors *xτhyp*, *rτhyp*, and *oτhyp* were all set to c(10,1), which are high precisions to reduce the width of the group distributions and thus to enforce the choice for a virus belonging to one of the two groups. Note that this implies that very high LRVs are unlikely.

Preprocessing of the data and calling JAGS was conducted in R (R Core Team 2019). Parameter estimates were obtained with 40,000 iterations of the Gibbs sampler, thinning every 10 iterations to decrease autocorrelations. The source code of the JAGS model is given in Appendix A3.

Postprocessing of the parameter estimates of the LRV model was conducted in Mathematica (v. 14.2, Wolfram Inc, IL). To all pairs of *rµ_s, g_* and *rτ_s,g_* per treatment, the parameters of two mixed normal distributions were estimated. These two distributions encompass variability and uncertainty of the group reductions. The distribution with the lowest mean reduction was labeled group 1, and the other distribution was labeled group 2.

### 2.6 QMRA

#### 2.6.1 Scenarios

QMRA encompassed exposure to pathogenic viruses from potable reuse of advanced treated wastewater to estimate risks of infection per person per year. For each facility, QMRA was conducted for adenovirus, enterovirus, and noroviruses GI and GII. For each of these four pathogenic viruses, the LRVs of those viruses were applied. Also, the LRVs of the associated group LRVs and the LRVs of crAssphage, PMMoV, and coliphages as surrogates for virus reduction were applied. The coliphages were characterized by plaque counts in contrast with genome copy counts of all other viruses. This resulted in a total of 72 QMRAs.

#### 2.6.2 Raw Wastewater Concentrations

A negative binomial distribution was fitted to the raw wastewater data (genome copy counts and sample volumes) for the four selected pathogenic viruses. From this, a Gamma distribution was derived as described by Schijven et al. (2011). To include parameter uncertainty, the algorithm of Metropolis and Hastings (Gilks and Roberts 1996) was used. See the Mathematica code in Appendix A4.

#### 2.6.3 Sensitivity of Detection, *S*

Distributions of sensitivity of detection *S* were fitted using the LRV model described in Section 2.5, with stage 1 for the raw data of the spiked viruses and stage 2 for the recovered raw data. HAdV‐4, CV‐B5, and MNV were surrogates for *S* of adenovirus, enterovirus, and norovirus GI and GII, respectively.

#### 2.6.4 LRV

Section 2.5 describes the LRV model. Predictive parameter samples were used to calculate LRV per virus and for the two groups per treatment stage and location. The total LRV, *TotalLRV*, was calculated by summing Monte Carlo (MC) LRV data of the subsequent treatment steps per facility.

#### 2.6.5 Potable Water Consumption

Potable water consumption in liters per person per day were obtained from USEPA (2006), which are included in QMRAspot (Schijven et al. 2011). The consumption data are lognormal distributed with a mean of −0.03598 and a standard deviation of 0.77218, corresponding to a mean of 1.3 L per person per day with a 95‐percentile range of 0.21−4.4 L.

#### 2.6.6 Infection Risk

From all distributions, an MC sample of size 10,000 was generated.

Exposure to the pathogenic viruses was given as the dose *D*, representing the number of ingested viruses per person per day and was calculated by multiplying the MC data of the source concentration, sensitivity of detection *S*, and total log reduction *TotalLRV*:

(13) $$D=C_{\mathrm{wastewater}}/\mathrm{Sx}10^{-\mathrm{TotalLRV}}\mathrm{xV}.$$

Infection risk per person per day was calculated by applying the hypergeometric dose−response relation with Beta‐distributed dose−response parameters (Teunis and Havelaar 2000; Teunis et al. 2008). The risk of infection for a specific dose was calculated as follows (Teunis et al. 2008):

(14) $$P_{\inf,\mathrm{person},\mathrm{day}}=1{-}_{1}F_{1}\left(\alpha ,\alpha +\beta ,-D\right),$$

where α and β are virus‐specific infectivity parameters, and *_1_F_1_* is the confluent hypergeometric function.

Infection risk per person per year was calculated by MC sampling from the daily infection risk:

(15) $$P_{\inf,\mathrm{person},\mathrm{year}}=1-\prod_{i=1}^{365}\left(1-P_{\inf,\mathrm{person},\mathrm{day}}\right).$$

The dose−response parameters *μw*, *μz*, *varw*, *varz*, and *cov* of the bivariate normal distribution for the selected pathogenic viruses are listed in Table 1. Using these parameter values, MC samples of pairs of infectivity parameters α and β were generated as described in detail by Teunis et al. (2020). The dose−response relationships for adenovirus and both noroviruses were based on genome copies from qPCR. The dose−response relationship of enterovirus was based on TCID_50_ counts of rotavirus. The tables also list the infection risk if D equals one genome copy of adenovirus and noroviruses GI and GII, and one infectious enterovirus particle.

**TABLE 1:** Dose−response parameters of the bivariate normal distribution.

Virus	*μw*	*μz*	*varw*	*varz*	*cov*	1 – *_1_F_1_* (*α*, *α* + *β*, – *1*)	Reference
Adenovirus	2.70	−0.0465	12.7	4.02	−0.0903	0.49	Teunis et al. (2016)
Enterovirus	0.571	−5.09	0.677	28.2	−2.73	0.41	Schijven et al. (2011)
Norovirus GI	−0.608	0.194	1.79	2.54	−1.03	0.27	Teunis et al. (2020)
Norovirus GII	−3.16	1.82	2.88	3.64	−2.01	0.077	Teunis et al. (2020)

#### 2.6.7 Required LRV

For each of the four pathogenic viruses at each facility, using Equations (13−15), given the concentration in raw wastewater and given the shape of the *TotalLRV* distribution, the required LRV was determined by shifting the mean *TotalLRV* such that the 95th percentile of the computed infection risk reached 10^−4^ per person per year.

## 3 Results

### 3.1 Sensitivity of Detection and Raw Wastewater Concentrations

**FIGURE A2:**
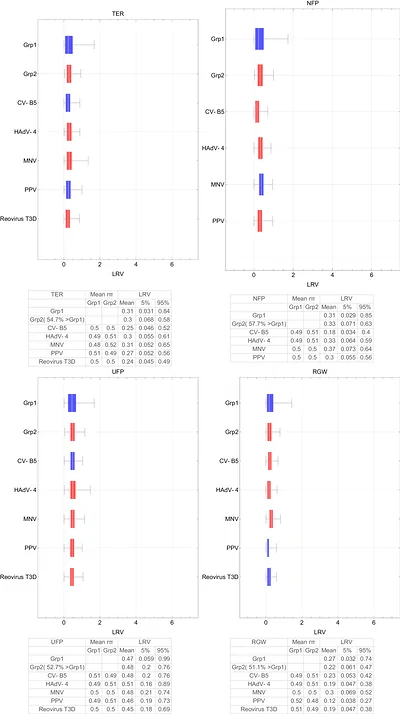
LRVs of viruses adjusted to sensitivity of detection (*S* = 10^−LRV^) in tertiary effluent (TER), ultrafiltration permeate (UFP), nanofiltration permeate (NFP), and recharge groundwater (RGW).

Figure A2 presents the LRVs of CV‐B5, HAdV‐4, MNV, PPV, and reovirus T3D adjusted to sensitivity *S* using box‐and‐whisker charts and tabled summary statistics (*S* = 10^−^ *^LRV^*) determined in tertiary effluent (TER), ultrafiltration permeate (UFP), nanofiltration permeate (NFP), and recharge groundwater (RGW). In all cases, the differences between groups 1 and 2 were not important and mean *rπ* values ranged from 0.49 to 0.51. This implies that *S* did not differ between the indicator viruses per type of effluent, which may be obvious because all viruses were detected in exactly the same samples with the same detection method but varied between effluent types. For TER and NFP, mean *S* equaled to 10^−0.3^ = 50%. For UFP, mean *S* equaled to 10^−0.5^ = 30%. For RGW, mean *S* equaled to 10^−0.2^ = 60%. The LRV data of TER were assumed to apply to *S* of adenovirus, enterovirus, and norovirus GI and GII, respectively, in raw wastewater of facilities A, B, and D.

**FIGURE 1:**
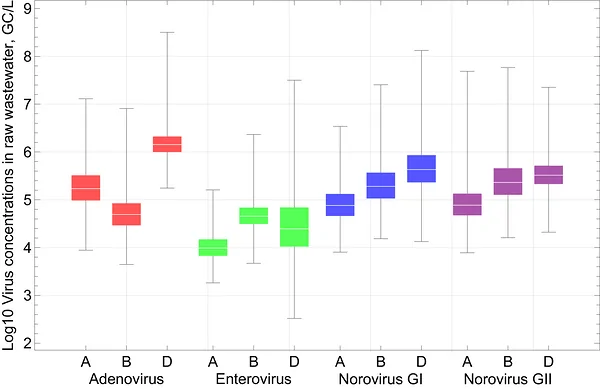
Distributions of the log_10_ of the raw wastewater concentrations corrected for sensitivity of detection at facility A (*N* = 14), B (*N* = 9), and C (*N* = 3).

**FIGURE A3:**
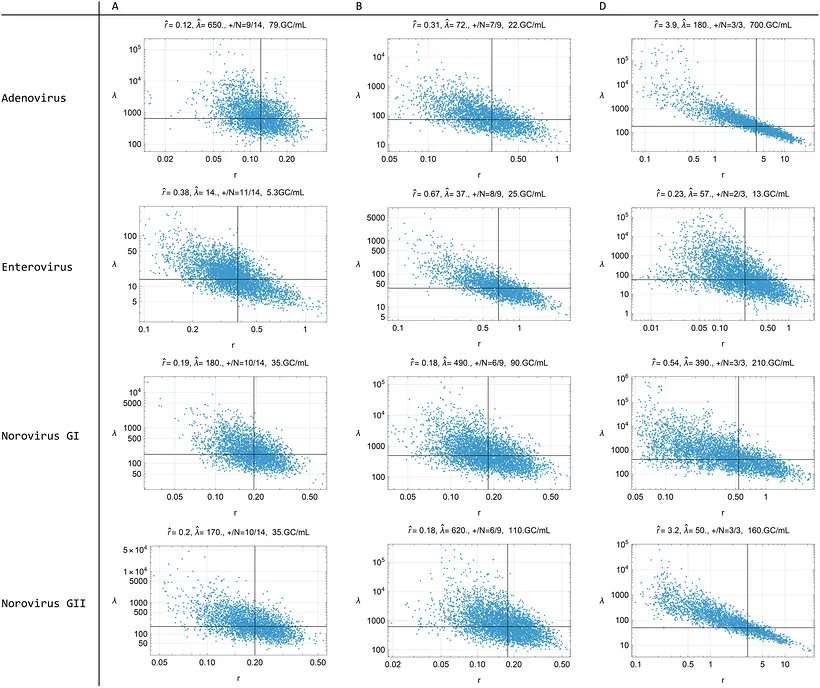
Distributions of parameters *r* and *λ* of the gamma‐distributed concentrations of adenovirus, enterovirus, norovirus GI, and norovirus GII in raw wastewater at facilities A, B, and D.

Figure A3 shows scatterplots of parameter estimates *r* and *λ* of the Gamma distributed concentrations of the selected pathogenic viruses at facilities A, B, and D. Figure 1 shows the box‐and‐whisker charts of the Gamma distributed concentrations on log_10_ scale of the selected pathogenic viruses at facilities A, B, and D corrected for *S*. The summary statistics are presented in Table A2. All concentrations were around 10^4^–10^5^ GC/L.

### 3.2 LRV Distributions

**FIGURE A4:**
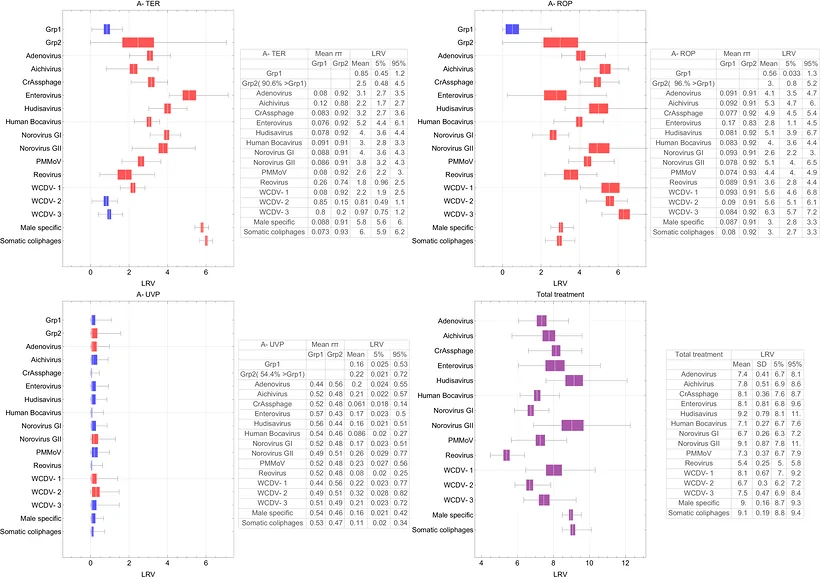
LRVs of all 15 viruses at facility A.

**FIGURE A5 risa70343-fig-0008:**
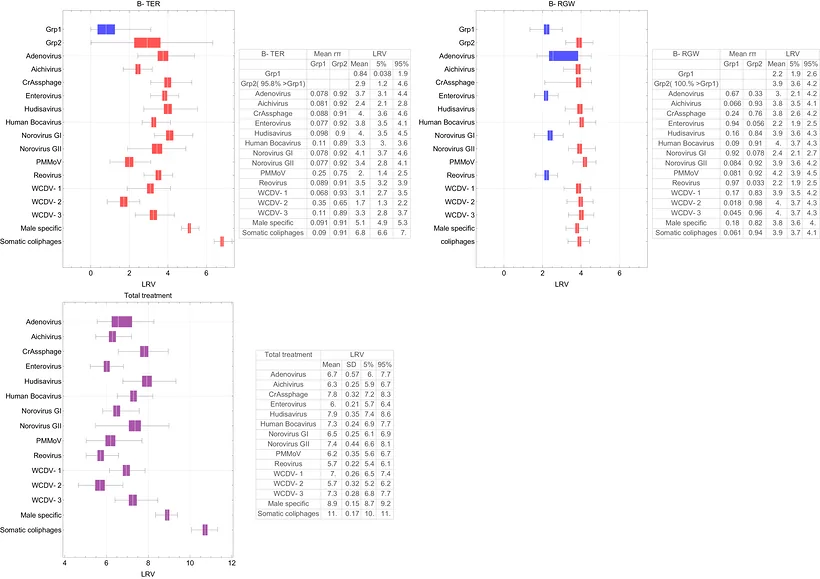
LRVs of all 15 viruses at facility B.

**FIGURE A6:**
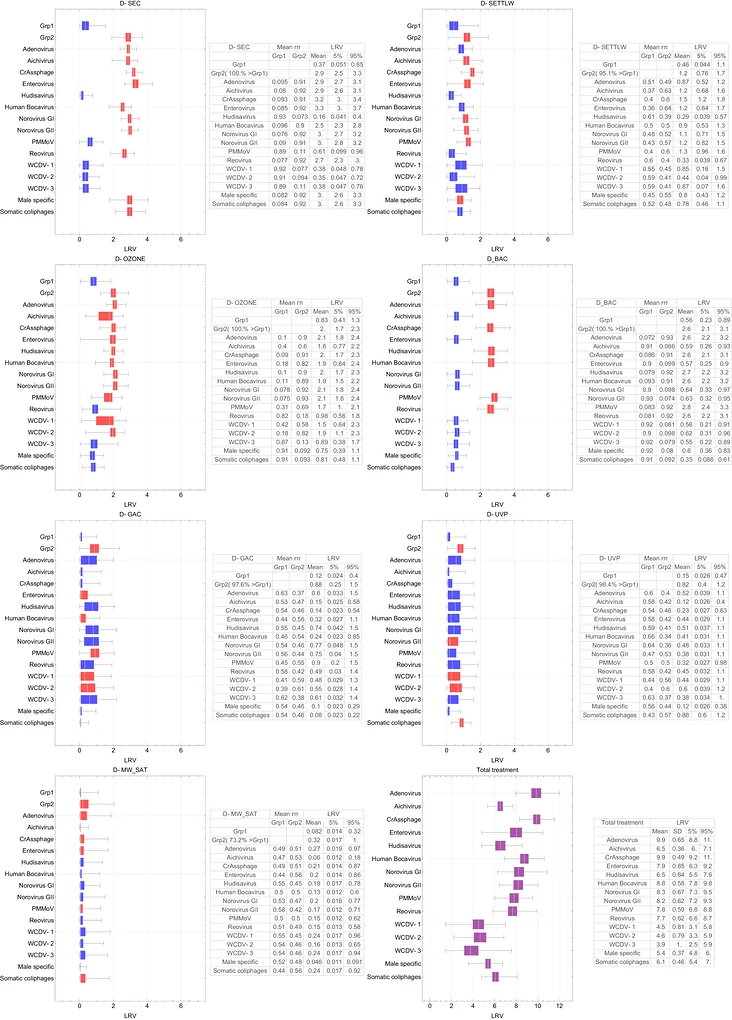
LRVs of all 15 viruses at facility D.

Table 2 summarizes LRV group statistics from the details on LRV distributions per facility, treatment, and virus or virus group as box‐and‐whisker charts in Figures A4−A6, each with tabled statistics. Table 2 also lists to which group a virus is classified.

**TABLE 2:** Group LRVs and classification of the 15 viruses over the two groups.

Treatment steps of facilities A, B, and D	% of LRVs of Group 2 > Group 1	Mean LRV 5%−95% LRV		Virus classification	
		Group 1	Group 2	Group 1	Group 2
A‐TER	90.1%	0.86 0.49–1.2	2.5 0.46–4.5	12, 13	1, 2, 3, 4, 5, 6, 7, 8, 9, 10, 11, 14, 15
A‐ROP	94.3%	0.56 0.038–1.3	3.0 0.55–5.3	—	1, 2, 3, 4, 5, 6, 7, 8, 9, 10, 11, 12, 13, 14, 15
A‐UVP	55.9%	0.16 0.024–0.53	0.23 0.021–0.72	2, 3, 4, 5, 6, 7, 9, 10, 13, 14, 15	1, 8, 11, 12
B‐TER	96.2%	0.85 0.037–1.9	3.0 1.3–4.6	—	1, 2, 3, 4, 5, 6, 7, 8, 9, 10, 11, 12, 13, 14, 15
B‐RGW	100%	2.2 1.9–2.5	3.9 3.6–4.2	1, 4, 7, 10	1, 2, 3, 5, 6, 8, 9, 11, 12, 13, 14, 15
D‐SEC	100%	0.38 0.051–0.85	2.9 2.5–3.3	5, 8, 10, 11, 12	1, 2, 3, 4, 6, 7, 9, 13, 14, 15
D‐SETTLW	95.5%	0.46 0.046–1.1	1.2 0.77–1.6	1, 5, 6, 10, 11, 12, 13, 15	2, 3, 4, 7, 8, 9, 14
D‐OZONE	100%	0.83 0.39–1.3	2.0 1.7–2.3	10, 13, 14, 15	1, 2, 3, 4, 5, 6, 7, 8, 9, 11, 12
D‐BAC	100%	0.57 0.23–0.91	2.6 2.1–3.1	2, 4, 7, 8, 11, 12, 13, 14, 15	1, 3, 5, 6, 9, 10
D‐GAC	97.4%	0.12 0.024–0.39	0.89 0.24–1.5	1, 2, 3, 5, 7, 8, 10, 13, 14, 15	4, 6, 9, 11, 12
D‐UVP	98.6%	0.14 0.027–0.45	0.82 0.41–1.2	1, 2, 3, 4, 5, 6, 7, 9, 10, 13, 14	8, 11, 12, 15
D‐MW_SAT	74.6%	0.081 0.014–0.32	0.33 0.017–1.0	5, 6, 7, 8, 10, 11, 12, 13, 14	1, 2, 3, 4, 9, 15

Viruses: 1. Adenovirus; 2. Aichivirus; 3. CrAssphage; 4. Enterovirus; 5. Hudisavirus; 6. Human Bocavirus; 7. Norovrius GI; 8. Norovirus GII; 9. PMMoV; 10. Reovirus; 11. WCDV‐1; 12. WCDV‐2; 13. WCDV‐3; 14. Male‐specific; 15. Somatic coliphages (Hudisavirus, WCDV‐1, WCDV‐2, WCDV‐3 are CRESS viruses).

The LRVs of group 2 are for 90%−100% of the cases higher than those of group 1, except for A‐UVP (56%) and D‐MW_SAT (75%), implying clear group separation for most treatment steps.

Mean *rπ* values of A‐TER, A‐ROP, B‐TER, B‐RGW, D‐SEC, D‐OZONE, and D‐BAC were between 0.74 and 0.9 for the associated group, implying that the probability of the LRVs of the viruses being assigned to that group was high. Mean *rπ* values of A‐UVP, D‐SETTLW, D‐GAC, D‐UVP, and D‐MW‐SAT were mostly near 0.4−0.6, implying that the LRVs of those viruses were not strongly part of either group.

The largest variation in LRVs between viruses and virus groups was seen in the first treatment steps. This variation was still large at the second treatment step of facility A. At facility A, sample series were the longest (*N* = 14). From the third treatment step of facility A, and from the second treatment steps of facilities B and D, differences in LRV among viruses were smaller. This was especially apparent at facility D with the shortest sample series (*N* = 3) and relatively highest proportion of nondetects. The combination of fewer samples and more nondetects made it difficult to discern differences in LRVs between viruses.

At the first treatment step of facilities A and B, LRVs of the male‐specific and somatic coliphages were 2–4 log_10_ higher than the LRVs of most other viruses, but in the following treatment steps, LRVs of both coliphages were like those of the other viruses. At the first treatment of facility D, LRVs of both coliphages appeared to be like those of most of the other viruses, possibly due to the low number of samples (*N* = 3), of which a large majority consisted of nondetects.

The studied viruses can also be grouped according to their genome type (Betancourt 2023):

‐ dsDNA: Adenovirus and CrAssphage.
‐ dsRNA: Reovirus.
‐ ssDNA: Hudisavirus, human bocavirus, and WCDV‐ 1, 2, and 3.
‐ ssRNA: Aichi virus, enterovirus, norovirus GI and GII, and PMMoV.
‐ Somatic coliphages can have either ssDNA or dsDNA. Male‐specific coliphages can have dsDNA, ssDNA, or ssRNA.

The LRV groups could not be associated with these virus genome groups, because the virus genome types seemed to be randomly dispersed over the LRV groups. All the studied viruses were nonenveloped. As with genome, the LRV groups could not be associated with virus size and isoelectric point either (see Table A5).

### 3.3 Total LRV

**FIGURE 2:**
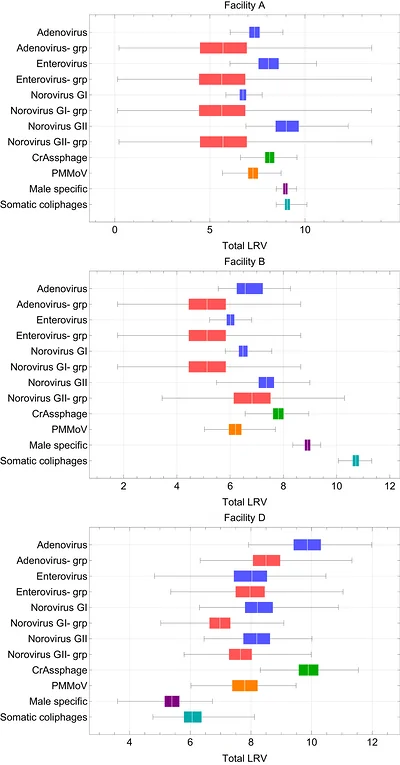
Distributions of total LRVs per selected pathogenic viruses and per facility based on the LRVs of those viruses (blue) and the associated group (grp, red). Results for crAssphage (green), PMMoV (orange), male‐specific (purple), and somatic coliphages (cyan) as indicators for virus reduction are also included.

Figure 2 shows the distributions of total LRVs of the selected pathogenic viruses per facility based on the LRVs of (1) those viruses, (2) the associated reduction group, (3) crAssphage, (4) PMMoV, (5) male‐specific, and (6) somatic coliphages as indicators for virus reduction. Table A3 provides the summary statistics. Mean total LRV or all individual viruses for facility A ranged from 5.6 to 9.2 log_10_, for facility B from 5.7 to 11, and for facility D from 5.4 to 9.9 log_10_.

At facility A, the group LRVs were equal for the four pathogenic viruses. Mean group LRVs were about 2–4 log_10_ lower than the corresponding single virus LRVs. The latter ranged from 6.7 to 9.1 log_10_. Mean LRVs of norovirus GII and both bacteriophages were estimated to be the highest.

At facility B, the group LRVs were equal for the four pathogenic viruses, except for norovirus GII, which were 1.7 log_10_ higher. Group LRV distributions were also generally wider than those of the single viruses. Mean group LRVs were 0.6–1.6 log_10_ lower than the corresponding single virus LRVs. The latter ranged from 6.1 to 11 log_10_. Mean LRV of somatic coliphages was the highest.

At facility D, the mean group LRVs ranged from 7.0 to 8.5 log_10_. All LRV distributions were similarly wide. Mean group LRVs were about −0.1 to 1.4 log_10_ lower than the corresponding single virus LRVs. The latter ranged from 5.4 to 9.9 log_10_. Mean LRVs of adenoviruses were estimated to be the highest.

At all facilities, LRVs of crAssphage were similar to or higher than those of the four pathogenic viruses, except at A for those of norovirus GII. At all facilities, LRVs of PMMoV were less than or like those of the four pathogenic viruses. Note that the number of nondetect samples of those two surrogate viruses was like that of the four pathogenic viruses (see Table A1).

At facility A, total LRVs of male‐specific and somatic coliphages were similarly high as those of norovirus GII. At facility B, somatic coliphages had the highest LRVs, followed by those of male‐specific coliphages. Note that the data of these two coliphages contained the fewest samples with nondetect results. Also, at facilities A and B, total LRVs of the two coliphages were 2–4 log_10_ higher than those of all other viruses. In contrast, total LRVs of those coliphages were relatively low at facility D. Possibly due to the sparse data of mostly nondetect LRVs of the other viruses at facility D may be overestimated.

### 3.4 Infection Risks

**FIGURE 3:**
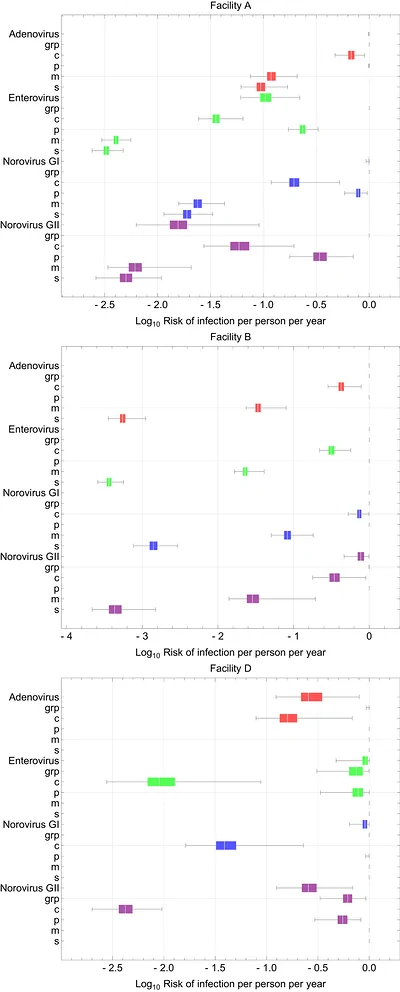
Distributions of log_10_ of infection risk per person per year for adenovirus (red), enterovirus (green), norovirus GI (blue), and norovirus GII (purple) at each facility based on the LRVs of those viruses and of the associated group (grp). Distributions are also given for crAssphage (c), PMMoV (p), male‐specific (m), and somatic coliphages (s) as indicators for virus reduction.

Figure 3 shows the distributions of risk of infection per person per year for each selected pathogenic virus and per facility based on the LRVs of those viruses and the associated group, as well as on the surrogate viruses. Table A4 provides the summary statistics.

At all facilities, for all viruses, distributions of the infection risks were higher than 10^−4^ per person per year. Except for norovirus GII at facility A, all infection risk distributions based on the LRVs of those viruses and the associated group fell between 0.1 and 1 per person per year.

The mean risks based on the LRVs of crAssphage and PMMoV varied between 0.01 and 1 at all facilities for all the selected pathogenic viruses. Except the mean risks based on LRVs of crAssphage were estimated to be lower for enterovirus and norovirus GI and GII.

The mean risks based on the LRVs of male‐specific and somatic coliphages varied between 10^−4^ and 0.1 at facilities A and B, but were 1 at facility D for all of the selected pathogenic viruses.

### 3.5 Required Total LRV

Table 3 shows required and achieved mean total LRVs and their differences given the concentrations of the four pathogenic viruses in raw wastewater at facilities A, B, and D and given the distributions of total LRVs in order not to exceed a risk of infection of 10^−4^ per person per year (95%). The mean required total LRVs varied between 11.4 and 13.4 log_10_, and the mean achieved total LRVs varied between 6.0 and 9.9 log_10_. In general, achieved total LRVs were 2.5–5.6 log_10_ too low to reach viral potable water quality.

**TABLE 3:** Mean log_10_ of required (*μ_req_*) and achieved (*μ_ach_*) total LRV and their difference.

	Facility A			Facility B			Facility D		
	*μ_req_*	*μ_ach_*	*μ_req_ − μ_ach_*	*μ_req_*	*μ_ach_*	*μ_req_ − μ_ach_*	*μ_req_*	*μ_ach_*	*μ_req_ − μ_ach_*
Adenovirus	12.5	7.4	5.1	11.8	6.7	5.1	13.4	9.9	3.5
Enterovirus	11.3	8.1	3.2	11.3	6.0	5.3	12.9	7.9	5.0
Norovirus GI	11.5	6.7	4.8	12.1	6.5	5.6	13.2	8.3	4.9
Norovirus GII	11.6	9.1	2.5	11.9	7.4	4.5	12.0	8.2	3.8

## 4 Discussion

### 4.1 LRV Model

Detection methods, like dPCR, allow analysis of (un)treated wastewater for a plethora of pathogenic viruses, which may provide a better estimation of treatment efficiency because not all viruses occur at the same frequency and levels. Grouping of log reductions is important for risk assessment because grouping shows how much LRVs may vary in natural conditions, among viruses, and treatment processes/locations. Grouping or pooling of log reductions enables representation of variability in pathogen reduction across viruses and treatment conditions, which is critical for bounding risk estimates in QMRA (e.g., Petterson and Stenström 2015; Tchobanoglous et al. 2022).

For all treatments, except for A‐RGW and D‐MW_SAT, the two LRV distribution groups were found to be different. The LRV model can deal with more than two groups, but given the data limitations, differentiation into three or more groups could not be made. Also, with sparse data and a majority of nondetects as observed in the later treatment steps, especially at facility D, variation in LRV among viruses could not be discerned. The observed differences in sampling frequency and duration among the facilities were primarily due to logistical and operational constraints rather than a deliberate experimental design. Specifically, longer sampling series were available for facilities A (14 samples collected monthly) and B (nine samples), whereas facility D was limited to three sampling events over the study period.

These differences do not introduce systematic bias in the estimation of treatment performance but rather affect the precision of the estimated LRVs. Facilities with more extensive sampling provide more precise estimates, while those with fewer samples result in greater uncertainty and wider ranges of plausible values.

Importantly, the Bayesian modeling framework integrates information across viruses, treatment steps, and facilities. This hierarchical structure allows estimates from data‐limited conditions to be informed by better‐sampled conditions, thereby stabilizing the results. Consequently, while reduced sampling density (as in facility D) makes it more challenging to distinguish differences among viruses or treatment steps, it does not inherently alter the overall estimates of treatment performance.

This interpretation is consistent with our observations that increased nondetects and limited sample size reduce the ability to resolve variability among viruses without indicating a systematic change in treatment efficacy. The lower LRVs observed in later treatment steps are, therefore, more likely related to the higher proportion of nondetects and persistence of more resistant viruses.

### 4.2 LRV Indicators

LRVs of the male‐specific and somatic coliphages were 2–4 log_10_ higher than the LRVs of most other eukaryotic viruses only at the first treatment step at facilities A and B, but not at facility D due to the low number of samples and mostly nondetects. For all viruses, except for coliphages, genome copies still contained in virus particles were counted. Free DNA and RNA or nonencapsidated genomes were eliminated by nuclease treatment prior to PCR. Therefore, their LRVs represent removal of virus particles. In contrast, the male‐specific and somatic coliphage counts were derived from infectious virus particles estimated by plaque assays. Therefore, their LRVs represent loss of infectivity, which can be due to removal of virus particles but also to damage of the virus capsid only. Loss of infectivity (inactivation) is likely to occur for all viruses along the treatment chain, and, apparently, mostly in the first treatment step. At facilities A and B, biologic activity in the first treatment step may have been responsible for much of the infectivity loss. However, loss of infectivity is not seen by counting genome copies enclosed in virions. The facts that the concentrations of virions of male‐specific and somatic coliphages in raw wastewater were higher than concentrations of the other viruses, that male‐specific and somatic coliphages were the least hampered by nondetects, that infectious virions were detected, albeit in a bacterial model system, and that their LRVs in the later treatment steps were similar to those of the other viruses, strongly indicate their usefulness as indicators for virus removal. Probably male‐specific coliphages are preferred over somatic coliphages, because the former is more specific to fecal contamination sources and the latter is more ubiquitous (Ballesté et al. 2021; Jofre et al. 2016). Choosing for male‐specific coliphage over somatic coliphage appeared also more conservative, but this may be facility‐specific.

CrAssphage and PMMoV suffered from as many nondetects as the other viruses enumerated by dPCR, and their possible loss of infectivity was not monitored either. Therefore, they did not seem to offer an advantage over the use of enumeration by dPCR of pathogenic viruses. Overall, LRVs of PMMoV were less than those of crAssphage. Both LRV distributions fell between those of the other viruses most of the time. In this sense, crAssphage, which is among the most abundant human‐associated viruses found in the human gut (Koonin and Yutin 2020), would be a more conservative indicator for virus reduction than PMMoV. If choosing one of the pathogenic viruses as an indicator for virus reduction, one should look at the lowest proportion of nondetects in combination with high infectivity. Adenovirus fits this profile best, and recent studies have already recognized its value as a reference pathogen when assessing unit process reduction credits for potable and nonpotable water reuse (Jahne et al. 2024).

The LRV groups could not be associated with the genome types, sizes, and isoelectric points of the studied viruses. Several explanations for not finding such an association may exist. A particular virus property may simply not play a role in a treatment process. It may be that a small fraction of the water bypassed treatment, in which case the same fraction of viruses was not treated and, consequently, LRVs were similar for all viruses. Viruses like adenovirus, enterovirus, norovirus, and coliphages form heterogeneous groups that vary in particular virus properties. The data did not allow discerning an association of virus property with reduction, for example, if data were too sparse and/or consist of many nondetects.

If a clear association would be identified between virus properties and its reduction in a treatment process, then the group LRV could be used to predict reduction of any virus with these properties. This implies that the group reductions found in this study cannot be used as indicators of reduction of viruses with the same genome type, size, and/or isoelectric point.[Fig risa70343-fig-0008]

### 4.3 QMRA

At all facilities, for all viruses, distributions of the infection risks were higher than 10^−4^ per person per year, and, even, of the 72 QMRA scenarios, 24 infection risks were equal to 1. This would imply that the finished water is not suitable for direct potable reuse.

However, the risks may have been overestimated, for which there are the following indications:

– At A‐TER and B‐TER, LRVs of the bacteriophages were 2–4 log_10_ higher than those of the other viruses, possibly due to loss of infectivity that was not picked up by dPCR for the other viruses. This indicates overestimation of infection risks by 2–4 log_10_.
– At facility D, a sample of each of RAW, SEC, and SETTL_W was analyzed for bacteriophages using plaque counting as well as dPCR. The concentrations are presented in Table 4. It appeared that in raw wastewater, concentrations in PFU/L were 210 and 54 times higher than the corresponding concentrations in GC/L. This can be explained by inhibition of the PCR reactions in the raw wastewater. Also, the probes for detecting the coliphages were more specific than classical plaque counting. For example, the probe for somatic coliphages was based on the sequence of ɸX174. This coliphage is known to be environmentally persistent (Bertrand et al. 2012). Classical plaque counting may encompass detection of less persistent coliphages. In contrast, concentrations in treated wastewater in PFU/L were 3.1, 170, 940, and 160 times lower than the corresponding concentration in GC/L. These numbers are single estimates, and therefore, only indicative, but they do support overestimation of the infection risks by 2–4 log_10_.
– Schijven et al. (2019) estimated the fraction of infectious adenoviruses enumerated by integrated cell culture PCR (ICC‐PCR) among adenoviruses enumerated by most probable number PCR (mpnPCR) and found a mean −log_10_ fraction of 3.2 (= 1/1700, 95% = 2.8 and 5% = 4.1). They did the same for rotavirus ICC‐PCR and mpnPCR data from Rutjes et al. (2009) and estimated a mean −log_10_ fraction of 2.0 (= 1/100, 95% = 1.4 and 5% = 5.2). Likewise, for enterovirus data by cell culture and mpnPCR from Lodder et al. (2015), a mean −log_10_ fraction of 1.4 (= 1/25, 95% = 0.76 and 5% = 4.7) was revealed. All these data were from intake surface waters at drinking water treatment plants. Rotavirus was enumerated in samples from the river Meuse, whereas adenovirus was enumerated in water from a storage reservoir fed with water from the river Meuse. Residence time in the storage reservoirs is 5 months on average. The detected adenoviruses might, therefore, have been longer in the environment following discharge with wastewater than the detected rotavirus, and, hence, there was more time for virus inactivation.
– Another comparison between qPCR and tissue culture enumeration was made by de Roda Husman et al. (2009), where inactivation of poliovirus‐1 and a coxsackievirus B4 was monitored for a period of 1 year in artificial groundwater and artificial surface water at 4°C and at 22°C. The fraction of infectious virus was initially about 1/100 and decreased to 1/10,000 or less depending on virus type, matrix, temperature, and time.

**TABLE 4:** Concentrations of coliphages as determined by dPCR (GC/L) and by classical plaque counting (PFU/L).

Stage	Phage	GC/L	PFU/L	PFU/GC
RAW	Somatic coliphage	1.2×10^5^	2.5×10^7^	2.1×10^2^ = 1/(4.8×10^−3^)
	Male‐specific phage GII	2.6×10^5^	1.4×10^7^	5.4×10^1^ = 1/(1.9×10^−2^)
SEC	Somatic coliphage	9.1×10^0^	2.9×10^0^	3.1×10^−1^ = 1/(3.1×10^0^)
	Male‐specific phage GII	1.5×10^2^	1.6×10^−1^	1.1×10^−3^ = 1/(9.4×10^2^)
SETTL_W	Somatic coliphage	1.2×10^2^	7.1×10^−1^	5.9×10^−3^ = 1/(1.7×10^2^)
	Male‐specific phage GII	2.7×10^1^	1.7×10^−1^	6.3×10^−3^ = 1/(1.6×10^2^)

Low ratios of infectious virions to genome copies can be caused by the presence of noninfectious particles with genomes that harbor lethal mutations or that have been damaged during growth or purification. Additionally, not all viruses in a preparation are capable of initiating infection because of the complexity of the infectious cycle (Genoyer and López 2019; Ke et al. 2013; Klasse 2015). Moreover, not all viruses replicate in the same cell line, and thus multiple cell lines may be required for detection of the wide range of infectious viruses present at specific levels of wastewater treatment (Gerba and Betancourt 2019).

Human challenge studies use inocula prepared from clinical samples and not from the environment. Adenoviruses, enteroviruses, and norovirus GI appear highly infectious, and it can easily be surmised that in the data from which the dose−response relationships were developed, the fraction of genome copies that were infectious virus particles was high, for example, more than 0.1. Norovirus GII is one order of magnitude less infectious.

Assuming a 3 log_10_ overestimation of the infection risks based on LRVs of the pathogenic viruses, the 95‐percentiles of the infection risks would still exceed 10–^4^ per person per year, except for norovirus GII at facility A. The 2–4 log_10_ overestimation should approximately be covered when using the somatic coliphages as an indicator for virus reduction, because of those reductions of infectious virus particles were measured, and those reductions were the highest. Risks based on LRVs of the somatic coliphages still fail 2–4 log_10_ short at facility A and 0.6–1.4 log_10_ at facility B (Table A4). It seems plausible that total LRVs at facility D were underestimated due to the limitation of the data.

Assuming overestimation of risks by 3 log_10_, mean total LRVs of 8.3–10.4 log_10_ might suffice. What determines the required LRV is the virus concentration in the raw wastewater and the infectivity of a virus as described by the dose−response relationship. Note that for raw wastewater concentrations of 10^4^–10^5^ GC/L, as found here for the selected pathogenic viruses, and a detection limit in the order of 1 per 1000 L, at total LRV of 7–8 log_10_, can be demonstrated. The concentrations of the bacteriophages were higher, in the order of 10^6^–10^8^ PFU/L, and thus allow demonstration of a total LRV of 9–11 log_10_. The fraction of infectious virus particles that formed the basis for the dose−response relationship may well differ from that in treated wastewater. Obviously, there is a need to reduce uncertainties in this regard by comparing genome copy counts with infectious units of viruses for which detection by tissue culture is possible, like for adenoviruses (Schijven et al. 2019). As long as this uncertainty remains, one may accept possible overestimation of risk to stay on the safe side.

## 5 Conclusions

The LRV model enabled estimation of LRVs for viruses from treatment data with many nondetects. The grouping of viruses with similar LRVs enabled more robust and less uncertain LRV estimates for single viruses. As shown for facilities A and B with series of 14 and nine samples, this worked well, although differences in LRVs between viruses diminished in later treatment steps. At facility D, with a series of three samples and a much longer treatment train, and consequently, mostly nondetects, LRVs were possibly overestimated. Therefore, longer sample series would allow better estimations.

Two distinct groups of LRVs of viruses could be identified. Mean total LRVs ranged from 5.4 to 11 log_10_. Required mean total LRVs for the four pathogenic viruses ranged from 11.3 to 13.4 log_10_.

At all facilities, for all four viruses, distributions of the infection risks were higher than 10^−4^ per person per year, implying that the finished water may not be safe to drink even when accounting for an overestimation of risk 2–4 log_10_.

Both male‐specific and somatic coliphages, detected by classical enumeration of infectious virions, are recommended as indicator viruses. Adenovirus was found to be the best pathogenic virus indicator for virus reduction due to its lowest proportion of nondetects in combination with high infectivity.

## Conflicts of Interest

The authors declare no conflicts of interest.

## Supporting information




**Supporting File**: risa70343‐supp‐0001‐SuppMat.xlsx

Supporting File: risa70343‐supp‐0002‐SuppMat.xlsx

## Appendix A

### A1 Tables

**TABLE A1:** Overview of numbers of samples (*N*) per facility, numbers of nondetects (ND), and numbers of samples were not used to produce a count (NA) per treatment step.

	Facility A, *N* = 14								Facility B, *N* = 9					
	SEW	TER	ROP	UVP	SEW	TER	RGW							
	ND	NA	ND	NA	ND	NA	ND	NA	ND	NA	ND	NA	ND	NA
Adenovirus	5	0	6	0	13	0	13	0	2	0	6	0	18	0
Aichivirus	4	0	4	0	13	0	14	0	2	0	2	0	16	0
CrAssphage	3	0	2	0	12	0	10	0	0	0	3	0	16	0
Enterovirus	3	0	14	0	14	0	14	0	1	0	7	0	16	0
Hudisavirus	5	0	9	0	14	0	14	0	0	3	4	3	12	6
Human Bocavirus	4	0	5	0	13	0	12	0	2	0	4	0	18	0
Norovirus GI	4	0	11	0	12	0	12	0	3	0	6	0	17	0
Norovirus GII	4	0	9	0	14	0	14	0	3	0	6	0	16	0
PMMoV	3	0	4	0	11	0	12	0	0	0	0	0	15	0
Reovirus	9	0	7	0	13	0	11	0	4	0	7	0	16	0
WCDV‐1	7	0	9	0	14	0	14	0	3	0	6	0	17	0
WCDV‐2	7	0	3	0	13	0	14	0	8	0	7	0	17	0
WCDV‐3	5	0	6	0	14	0	14	0	5	0	5	0	18	0
Male‐specific	0	1	1	1	8	1	9	1	0	1	0	1	10	2
Somatic coliphages	0	0	0	0	8	0	9	0	0	2	1	2	11	4

	Facility D, *N* = 3															
	SEW		SEC		SETTL_W		OZONE		BAC		GAC		UVP		MW_SAT	
	ND	NA	ND	NA	ND	NA	ND	NA	ND	NA	ND	NA	ND	NA	ND	NA
Adenovirus	0	0	0	0	0	0	1	0	3	0	3	0	3	0	3	0
Aichivirus	0	0	0	0	1	0	2	0	3	0	2	0	3	0	1	0
CrAssphage	0	0	0	0	0	0	1	0	3	0	3	0	2	0	3	0
Enterovirus	1	0	3	0	3	0	3	0	3	0	3	0	3	0	3	0
Hudisavirus	3	0	0	0	0	0	2	0	3	0	3	0	3	0	3	0
Human Bocavirus	0	0	0	0	0	0	1	0	3	0	3	0	3	0	3	0
Norovirus GI	0	0	0	0	0	0	3	0	3	0	3	0	3	0	3	0
Norovirus GII	0	0	0	0	0	0	3	0	3	0	3	0	3	0	3	0
PMMoV	0	0	1	0	1	0	2	0	3	0	3	0	3	0	3	0
Reovirus	2	0	3	0	2	0	2	0	2	0	3	0	3	0	3	0
WCDV‐1	3	0	2	0	3	0	3	0	3	0	3	0	3	0	3	0
WCDV‐2	3	0	3	0	2	0	2	0	3	0	3	0	3	0	3	0
WCDV‐3	3	0	0	0	3	0	3	0	3	0	3	0	3	0	3	0
Male‐specific	0	1	0	1	0	1	1	0	0	0	1	0	1	0	1	0
Somatic coliphages	0	1	0	1	0	1	0	2	0	0	0	0	0	0	0	3

**TABLE A2:** Summary statistics of the concentrations of adenovirus, enterovirus, norovirus GI, and norovirus GII in raw wastewater at facilities A, B, and D corrected for sensitivity of detection.

Virus	Facility	Mean	Median	SD	5%	95%
Adenovirus	A	5.3	5.2	0.42	4.7	6.0
	B	4.7	4.7	0.34	4.2	5.3
	D	6.2	6.2	0.25	5.8	6.6
Enterovirus	A	4.0	4.0	0.27	3.6	4.5
	B	4.7	4.7	0.27	4.3	5.1
	D	4.5	4.4	0.70	3.5	5.8
Norovirus GI	A	4.9	4.9	0.36	4.4	5.5
	B	5.3	5.3	0.43	4.7	6.1
	D	5.7	5.6	0.47	5.0	6.5
Norovirus GII	A	4.9	4.9	0.37	4.4	5.6
	B	5.4	5.4	0.45	4.8	6.2
	D	5.5	5.5	0.30	5.1	6.1

**TABLE A3:** Total LRVs.

	Facility A			Facility B			Facility D		
	Mean	5%	95%	Mean	5%	95%	Mean	5%	95%
Adenovirus	7.4	6.7	8.1	6.7	6.0	7.7	9.9	8.8	11
Adenovirus‐group	5.7	2.7	8.7	5.1	3.4	6.8	8.5	7.5	9.7
Enterovirus	8.1	6.8	9.6	6.0	57	6.4	7.9	6.3	9.2
Enterovirus‐group	5.6	2.7	8.7	5.1	3.4	6.8	8.0	6.9	9.2
Norovirus GI	6.7	6.3	7.2	6.5	6.1	6.9	8.3	7.3	9.5
Norovirus GI‐group	5.6	2.7	8.7	5.1	3.4	6.8	7.0	6.2	7.8
Norovirus GII	9.1	7.8	11	7.4	6.6	8.1	8.2	7.2	9.3
Norovirus GII‐group	5.7	2.7	8.7	6.8	5.1	8.5	7.7	6.8	8.6
crAssphage	8.1	7.6	8.7	7.8	7.2	8.3	9.9	9.2	11
PMMoV	7.3	6.7	7.9	6.2	5.7	6.7	7.8	6.8	8.8
Male‐specific	9.0	8.7	9.3	8.9	8.7	9.2	5.4	4.8	6.0
Somatic coliphages	9.1	8.8	9.4	11	10	11	6.1	5.4	7.0

**TABLE A4:** Log_10_ of risk of infection per person per year for each selected pathogenic virus at each facility based on the LRVs of those viruses, the associated group (grp), crAssphage (c), PMMoV (p), male‐specific (m), and somatic coliphages (s).

	Facility A			Facility B			Facility D		
	Mean	5%	95%	Mean	5%	95%	Mean	5%	95%
Adenovirus	0	0	0	0	0	0	−0.69	−0.80	−0.57
grp	0	0	0	0	0	0	0	0	0
c	−0.1	−0.2	0	−0.34	−0.43	−0.21	−0.92	−1.0	−0.82
p	0	0	0	0	0	0	0	0	0
m	−0.72	−0.97	−0.13	−1.5	−1.5	−1.3	0	0	0
s	−0.81	−1.1	−0.35	−3.2	−3.3	−3.1	0	0	0
Enterovirus	−0.96	−1.1	−0.8	0	0	0	−0.03	−0.10	0
grp	0	0	0	0	0	0	−0.15	−0.31	−0.02
c	−1.4	−1.5	−1.3	−0.53	−0.59	−0.46	−2.0	−2.3	−1.7
p	−0.62	−0.69	−0.54	0	0	0	−0.12	−0.24	−0.03
m	−2.4	−2.4	−2.3	−1.7	−1.7	−1.6	0	0	0
s	−2.5	−2.5	−2.4	−3.5	−3.5	−3.4	0	0	0
Norovirus GI	0	0	0	0	0	0	−0.04	−0.09	0
grp	0	0	0	0	0	0	0	0	0
c	−0.74	−0.84	−0.61	−0.10	−0.17	−0.04	−1.4	−1.6	−1.1
p	−0.12	−0.16	−0.07	0	0	0	0	0	0
m	−1.7	−1.7	−1.5	−0.97	−1.1	−0.69	0	0	0
s	−1.8	−1.8	−1.7	−2.7	−2.9	−2.5	0	0	0
Norovirus GII	−1.7	−2.0	−1.5	−0.09	−0.17	−0.027	−0.55	−0.71	−0.7
grp	0	0	0	0	0	0	−0.20	−0.31	−0.09
c	−1.2	−1.4	−0.92	−0.37	−0.53	−0.21	−2.3	−2.5	−2.0
p	−0.46	−0.60	−0.30	0	0	0	−0.23	−0.37	−0.09
m	−2.2	−2.3	−2.0	−1.4	−1.6	−1.2	0	0	0
s	−2.3	−2.4	−2.1	−3.2	−3.4	−2.9	0	0	0

**TABLE A5:** Properties of the studied viruses.

	Virus genome	Size (nm)	Isoelectric point
Adenovirus	dsDNA	90–100	4.5–6.75
Aichivirus	ssRNA	30–32	7.2
CrAssphage	dsDNA	75–80	NA
Enterovirus	ssRNA	30–32	4.5–8.3
Hudisavirus	ssDNA	15–25	NA
Human Bocavirus	ssDNA	23–25	7.4
Norovirus GI	ssRNA	NA	NA
Norovirus GII	ssRNA	27–40	5.5–6.0
PMMoV	ssRNA	18×300–310	3.7–3.8
Reovirus	dsRNA	70–90	3.9
WCDV‐1	ssDNA	15–25	NA
WCDV‐2	ssDNA	15–25	NA
WCDV‐3	ssDNA	15–25	NA
Male‐specific	ssRNA/ssDNA	21–30/4–12	3.9/2.7–5.3
Somatic coliphages	ssDNA/dsDNA	21–25/60–595	6.6–7.3

*Note*: Data taken from Table 1.3 in Betancourt et al. (2023).

### A3 JAGS Code

# JAGS Model for Poisson distributed counts (plaque counts, cytopathic effect units, dPCR genome copies)

model{

for(stage in 1:nstage){# start by estimating virus concentrations

for(virus in 1:nvirus){

for(obs in 1:nobs[stage, virus]){

n.obs[stage, virus, obs] ∼ dpois(exp(lc.obs[stage, virus, obs])*v.obs[stage, virus, obs])

lc.obs[stage, virus, obs] ∼ dnorm(lcmu[stage, virus, obs], lctau[stage, virus])

}}}

# JAGS Model for qPCR genome copies; replaces the code above

# model{

# for(stage in 1:nstage){# start by estimating virus concentrations

# for(virus in 1:nvirus){

# for(obs in 1:nobs[stage, virus]){

# x.cens[stage, virus, obs] ∼ dinterval(x[stage, virus, obs], xlim[stage, virus, obs])

# x[stage, virus, obs] ∼ dnorm(xmu[stage, virus, obs], xtau[stage, virus])

#}}}

# Following code the same for both models

for(stage in 2:nstage){# now determine pairwise reductions (robs)

for(virus in 1:nvirus){

for(obs in 1:nobs[stage, virus]){

xmu[stage, virus, obs] ← xmu[stage‐1, virus, obs]—robs[stage‐1, virus, obs]

robs[stage‐1, virus, obs] ∼

dnorm(red[stage‐1, virus], otau[stage‐1, virus])

} # and reduction by stage and virus type (red)

rred[stage‐1, virus] ∼

dnormmix(rmu[stage‐1, 1:ngroup], rtau[stage‐1, 1:ngroup],

rpi[stage‐1, virus, 1:ngroup])

red[stage‐1, virus] ← ifelse(step(rred[stage‐1, virus]‐inflec),

rred[stage‐1, virus],

inflec*exp(rred[stage‐1, virus]‐inflec))

rpi[stage‐1, virus, 1:ngroup] ∼ ddirch(alpha[stage‐1, 1:ngroup])

}

}

for(virus in 1:nvirus){# sample from (hyper)priors

for(obs in 1:nobs[1, virus]){

xmu[1, virus, obs] ∼ dnorm(cmu.hyp[virus, 1], cmu.hyp[virus, 2])

}

for(stage in 1:nstage){

otau[stage, virus] ∼ dgamma(otau.hyp[stage, 1], otau.hyp[stage, 2])

xtau[stage, virus] ∼ dgamma(xtau.hyp[stage, 1], xtau.hyp[stage, 2])

}

}

for(stage in 1:(nstage‐1)){

for(group in 1:ngroup){

rmu[stage, group] ∼ dnorm(rmu.hyp[stage, group, 1], rmu.hyp[stage, group, 2])

rtau[stage, group] ∼

dgamma(rtau.hyp[stage, group, 1], rtau.hyp[stage, group, 2])

}

}

}

### A4 Metropolis Hastings Parameter Estimation, Mathematica Code

USAGE

Estimate parameters r and lam of negative binomially distributed counts and volumes

The concentrations (counts per volume) are Gamma distributed with parameters r and lambda

PARAMETERS

Data = 1st column for counts and 2nd column for sample volumes

Mlefun = maximum loglikelihood function, in this case function fNegBin to find the values for r and λ

Likfun = likelihood function, in this case function NegativeBinomialLikelihood used for acceptance of a state of parameter values

Nit = number of iterations

Res(ults) = {gpars, mhpars, lobs, npos, ltr, rej, fconcs, rcf, pf2@tim[[1]], sig}

Gpars = estimates of r and λ using mlefun as initial parameter values (allows short burn in)

Mhpars = Markov Chain Monte Carlo values of r and λ

Lobs = length of total number of observations (data)

Npos = number of positive observations

Ltr = length of the trail (succesful iterations)

Rej = number of rejections

Fconcs = sorted tally of concentrations

Rcf = cumulative fconcs/ number of data

Pf2@tim[[1]] = iteration time

Sig = significance criterion for rejection

NOTES

Rejection between 23% and 50% is strict.

A burn in number of 100 is safe. A maximum likelihood estimate is made in advance.

A 95%‐contour plot corresponds to 5% deviance.

REFERENCE

Gilks, W. R., Richardson, S., & Spiegelhalter, D. J. (1996). Markov Chain Monte Carlo in practice. London: Chapman and Hall.

(*Metropolis‐Hastings algorithm*)

MetropolisHastingsParameterEstimation[data_, mlefun_, likfun_, nit_]: =

Block{sig, counts, volumes, obs, lobs, npos, fconcs, rcf, gpars, sigpars, state, trail, dstate, pi, tim, rej = 0, ltr, mhpars}

Counts = data[[All, 1]]; volumes = data[[All, 2]];

Obs = Transpose@{counts, volumes 1000};(*volumes set from L t mL; in L small numbers*) lobs = Length@obs;npos = Length@Select[data[[All, 1]], #>0&]; fconcs = Tally[Sort[data[[All, 1]]/data[[All, 2]]]];rcf = Accumulate[fconcs[[All, 1]]]/Length@data;

If[Total@obs[[All, 1]] = = 0, “Only non detects”, (*data as counts and volumes in pairs*) gpars = mlefun@obs;(*MLE estimate to start*)

Sig = .2;

Sigpars = Abs@state sig;

(*BURN IN 100 TO SET SIG*)

While[

(*Need between 23% and 50% rejections*)

Rej<23||rej>50,

Rej = 0;state = Log@gpars;trail = {state};

Table[

Dstate = Table[RandomReal@NormalDistribution[0, sigpars[[i]]], {i, Length@sigpars}];

If[(*Test for acceptance of new state*) RandomReal[]≤likfun[Exp[state+dstate], obs]/likfun[Exp@state, obs],

(*True = accepted, update state of parameters*)

State = state+dstate,

(*False = rejected, increase number of rejections*)

Rej = rej+1];

(*Extend trail*)

Trail = Append[trail, state], {100(*burn number*)}];

(*Adjust sig*)

Which[

Rej<23, sig = sig 2 (*too few rejections, increase sig*),

Rej>50, sig = sig/2 (*too many rejections, decrease sig*),

True, sig];

Sigpars = Abs@state sig;];

(*MCMC*)

Rej = 0;state = Log@gpars;trail = {state};

Pi = 0;

Monitor[tim = Timing@Table[

Pi++;

Dstate = Table[RandomReal@NormalDistribution[0, sigpars[[i]]], {i, Length@sigpars}];

If[(*Test for acceptance of new state*)

RandomReal[]≤likfun[Exp[state+dstate], obs]/likfun[Exp@state, obs],

(*True = accepted, update state of parameters*)

State = state+dstate,

(*False = rejected, increase number of rejections*)

Rej = rej+1];

Trail = Append[trail, state], {nit}], ProgressIndicator[pi, {0, nit}]];

Ltr = Length@trail;

Mhpars = Exp@Table[trail[[i]], {i, 1, ltr, 1+Round[ltr/(ltr‐rej)](*thinning*)}];

{gpars, mhpars, lobs, npos, ltr, rej, fconcs, rcf, pf2@tim[[1]], sig}]];
